# Nonsteroidal anti-inflammatory drug exposure and the risk of microscopic colitis

**DOI:** 10.1186/s12876-022-02438-z

**Published:** 2022-07-30

**Authors:** Eugene F. Yen, Daniel B. Amusin, Janet Yoo, Asantewaa Ture, Nicole M. Gentile, Michael J. Goldberg, Jay L. Goldstein

**Affiliations:** 1 Division of Gastroenterology, NorthShore University HealthSystem, Evanston, IL USA; 2 Rush University College of Nursing, Chicago, IL USA; 3 Northwestern University Feinberg School of Medicine, Chicago, IL USA

**Keywords:** Microscopic colitis, Collagenous colitis, Lymphocytic colitis, Non-steroidal anti-inflammatory drugs

## Abstract

**Background:**

Medication consumption has been suggested as a risk factor for microscopic colitis (MC), but studies of varying design have yielded inconsistent results. Our aim was to evaluate the association between medications and MC.

**Methods:**

A hybrid cohort of prospectively identified patients undergoing colonoscopy with biopsies for suspicion of MC (N = 144) and patients with MC enrolled within three months of diagnosis into an MC registry (N = 59) were surveyed on medication use. Medication use was compared between patients with and without diagnosis of MC by chi-squared test and binomial logistic regression adjusted for known risk factors of MC: age and gender.

**Results:**

In total, 80 patients with MC (21 new, 59 registry) were enrolled. Patients with MC were more likely to be older (*p* = 0.03) and female (*p* = 0.01) compared to those without MC. Aspirin and other non-steroidal anti-inflammatory drugs were more commonly used among patients who developed MC (*p* < 0.01). After controlling for age and gender, these medications remained independent predictors of MC with odds ratio for any non-steroidal anti-inflammatory drug use of 3.04 (95% CI: 1.65–5.69). No association between MC and other previously implicated medications including proton pump inhibitors and selective serotonin reuptake inhibitors was found.

**Conclusions:**

In this cohort of patients with chronic diarrhea, we found use of aspirin and non-steroidal anti-inflammatory drugs, but not other implicated medications to be associated with the development of MC. Whether these drugs trigger colonic inflammation in predisposed hosts or worsen diarrhea in undiagnosed patients is unclear. However, we feel that these findings are sufficient to discuss potential non-steroidal anti-inflammatory drug cessation in patients newly diagnosed with MC.

**Supplementary Information:**

The online version contains supplementary material available at 10.1186/s12876-022-02438-z.

## Background

Microscopic colitis (MC), delineated into the categories of lymphocytic colitis (LC) and collagenous colitis (CC), is a common cause of chronic watery diarrhea encountered in the clinical setting. The incidence of MC has risen dramatically over the past several decades and stabilized to levels equal or greater than that of Crohn’s disease and ulcerative colitis [1–4].

MC has been strongly associated with female gender, older age, and autoimmune disease. Furthermore, environmental factors such as smoking have also shown association with MC [2–15]. Of particular interest is the emerging entity of “medication induced MC.” Initial evidence for this phenomenon was offered by several large retrospective case–control studies utilizing national databases and smaller case series. These studies suggest varying risk of MC in patients taking non-steroidal anti-inflammatory drugs (NSAIDs), proton-pump inhibitors (PPIs), selective serotonin reuptake inhibitors (SSRIs), beta blockers, angiotensin-converting enzyme inhibitors (ACE-Is) and HMG-CoA reductase inhibitors (statins) [16–18]. However, these results were limited by their retrospective design, especially regarding over-the-counter medications such as NSAIDs and PPIs. Further, medications commonly cause diarrhea, particularly in older individuals where MC is more prevalent.

Further studies continued investigating the role of medications in MC while utilizing control patients evaluated by colonoscopy for diarrhea. However, they yielded mixed results, particularly with respect to the risk of NSAIDs and PPIs [19, 20]. Therefore, the purpose of the present study was to continue investigating the potential association between MC and medication use in patients being investigated for chronic diarrhea or recently diagnosed with MC.

## Methods

### Patient selection & data collection

We identified a hybrid cohort of patients consisting of those prospectively evaluated for chronic watery diarrhea and those previously enrolled in our center’s established MC registry. First, cases and controls were identified prospectively by identification of patients with a planned colonoscopy for evaluation of suspected MC. Patients exhibited chronic daily diarrhea (greater than or equal to 3 bowel movements per day) for greater than two months with a planned colonoscopy to specifically rule out MC or other causes of diarrhea. Patients with known cause of diarrhea such as *Clostridioides difficile* infection, inflammatory bowel diseases (ulcerative colitis and Crohn’s disease), malignancy or abnormal imaging results were excluded from the study.

Prior to the procedure, patients were surveyed at either clinic or bedside by a research coordinator using a script (Additional file 1) with questions regarding demographic information, frequency and duration of diarrhea, and use of the following medications: aspirin (81 mg or greater), other NSAIDs, PPIs, H2RAs, SSRIs, statins, ACE-Is, and angiotensin receptor blockers (ARBs, ARB data was not collected in 8 new MC, and 21 no MC patients due to study protocol revision). Patients were classified as current (greater than seven cigarettes per week for at least six months), former (quit smoking any time prior to MC diagnosis), or never smokers. As reported in previous studies, medication use was defined as “taking the medicine at least three times a week for at least two weeks” [12, 21].

After survey, colonoscopic evaluation was performed with biopsies taken from ascending, transverse, and descending segments of colon. Biopsies were assessed for MC by expert gastrointestinal pathologists and classified into CC or LC. CC was defined by the histologic criteria: thickness of the collagenous subepithelial table > 10 μm using an ocular micrometer, inflammation in the lamina propria consisting of mainly lymphocytes and plasma cells, lack of crypt architectural distortion, and regenerative-appearing changes in the surface and/or crypt epithelium. Histologic criteria for LC was defined by the following: intraepithelial lymphocytes > 20 per 100 epithelial cells in the subjective area of highest lymphocyte density, inflammation in the lamina propria consisting of mainly lymphocytes and plasma cells, and regenerative-appearing changes in the surface and/or crypt epithelium.

Additional patients with MC were identified from our center’s established MC registry. The registry consists of histologically verified patients diagnosed with MC at our health system since 2012, and follows patient history, potential offending medications, MC treatments, and outcomes. All patients enrolled into the registry from 2012 to 2020 whose data was collected within three months of diagnosis were entered into the present study. Medication use in this group of patients was collected in an identical standardized manner to those prospectively identified.

### Statistical analysis

Power analysis was conducted to determine a necessary sample size to achieve 80% power at a significance level of 0.05. Previously reported data on NSAID usage among MC (*P*_1_ = 0.46) and control patients (*P*_2_ = 0.22) indicated a necessary sample size of 62 patients per group [21]. Similar analysis regarding PPIs (*P*_1_ = 0.46, *P*_2_ = 0.14) estimated a sample size of 29 patients per group [18].

Significance testing for demographic differences and rate of medication use among registry and new MC cohorts, MC and no MC cohorts, and MC subtypes was conducted by Welch’s t-test, chi-squared test for homogeneity, and Fisher exact test. Consistency of the overall results was assessed through a subgroup analysis using identical tests that compared medication use among the two MC groups to the no MC group. Furthermore, to control for demographic differences among cohorts, binomial logistic regressions were modelled with parameters for age, gender, and medication use. Any missing data was excluded from analysis. All statistical tests were performed using R version 4.1.1[22].

### Ethical considerations

The present study was performed in accordance with the principles of the Declaration of Helsinki and approved by the Institutional Review Board at the NorthShore University HealthSystem Research Institute.

## Results

### Patient demographics

**Fig. 1:**
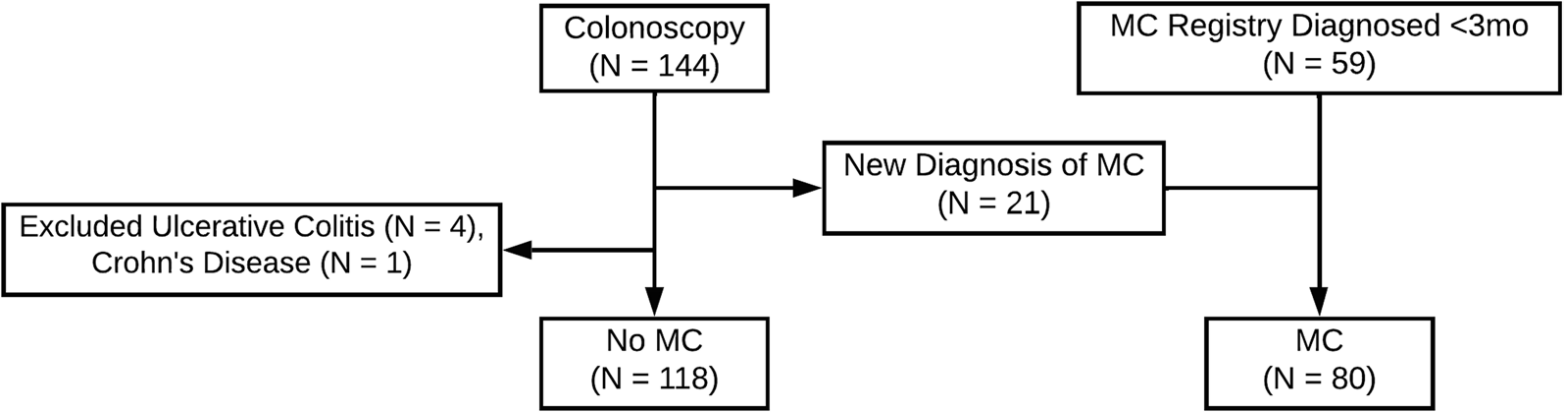
Cohort Selection. This flowchart illustrates the two sources of subjects included in the study. The no microscopic colitis (MC) group consisted of 118 patients, and MC group consisted of 80 patients with 21 newly diagnosed, and 59 identified within the MC registry

From 2012 to 2016, 144 patients underwent colonoscopic evaluation in accordance with the inclusion criteria. Of these, 123 patients were negative for MC, and 5 were excluded with newly diagnosed ulcerative colitis (4) and Crohn’s disease (1). MC was diagnosed in 21 new patients (6 CC, 15 LC), and an additional 59 patients were identified for inclusion from the MC registry (14 CC, 45 LC). This yielded final cohorts of MC and no MC with sizes 80 (20 CC, 60 LC) and 118 patients, respectively (Fig. 1).

Patient demographics are displayed in Table 1. No significant differences were observed between MC patients diagnosed at colonoscopy (“new MC”) and those from the MC registry group. Expected demographic variability was noted between MC and no MC cohorts in terms of gender and age. Females accounted for 81.3% of MC, and 66.1% of control patients (*p* = 0.01), and MC patients were on average 4.8 years older than those without MC with mean ages of 60.4 and 55.6 respectively (*p* = 0.03). Race/ethnicity, smoking history, and diagnosis of celiac disease were not found to differ significantly between study cohorts.

**Table 1:** Patient demographics

	Total (%)	New MC (%)	MC Registry (%)	*p*-val^a^	All MC (%)	No MC (%)	*p*-val^b^
Number of patients	198	21 (10.61)	59 (29.80)		80 (40.40)	118 (59.60)	
Female	140 (70.71)	16 (76.19)	49 (83.05)	0.49	65 (81.25)	78 (66.10)	0.01
Race/ethnicity							
Caucasian	179 (90.140)	18 (85.71)	57 (96.61)	0.14	75 (93.75)	104 (88.14)	0.24
Hispanic	5 (2.53)	2 (9.52)	1 (1.69)		3 (3.75)	2 (1.63)	
African American	4 (2.02)	0 (0.00)	1 (1.69)		1 (1.25)	3 (2.54)	
Asian	4 (2.02)	0 (0.00)	0 (0.00)		0 (0.00)	4 (3.25)	
Other/unknown	6 (3.03)	1 (4.76)	0 (0.00)		1 (1.25)	5 (4.07)	
Age (years), Mean ± Standard deviation	57.55 ± 15.27	59.05 ± 15.04	60.86 ± 14.42	0.63	60.38 ± 14.51	55.62 ± 15.53	0.03
Smoking status							
Current	20 (10.10)	2 (9.52)	10 (16.95)	0.70	12 (15.00)	8 (6.78)	0.13
Former	68 (34.34)	8 (38.10)	22 (37.29)		30 (37.50)	38 (32.20)	
Never	109 (55.05)	11 (52.38)	27 (45.76)		38 (47.50)	71 (60.20)	
History of celiac disease	2 (1.01)	0 (0.00)	1 (1.69)	1.00	1 (1.22)	1 (0.85)	0.78
Diagnosis							
Collagenous colitis		6 (28.57)	14 (23.73)	0.66	20 (25.00)	–	–
Lymphocytic colitis		15 (71.43)	45 (76.27)		60 (75.00)	–	

Demographic characteristics are listed, first comparing patients with newly diagnosed microscopic colitis (MC) at colonoscopy and those obtained from the MC registry (*p*-val^a^), followed by all MC patients compared to diarrheal controls negative for MC on colonoscopy (*p*-val^b^)

### Medication usage

**Fig. 2:**
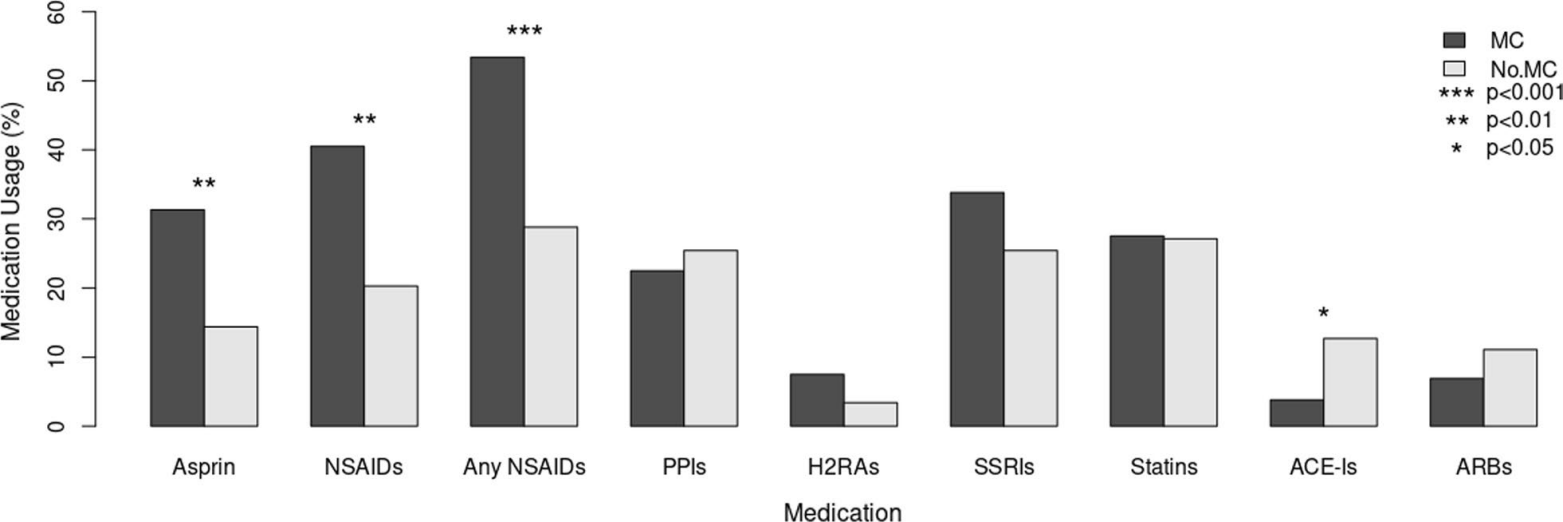
Proportion of Medication Usage Among MC and No MC Cohorts. The plot above illustrates the relative proportion of medication users between microscopic colitis (MC) and no MC cohorts. MC was significantly associated with increased use of aspirin (*p* < 0.05), other non-steroidal anti-inflammatory drugs (*p* < 0.05) or both (*p* < 0.001) and decreased use of angiotensin converting enzyme inhibitors (*p* < 0.05). NSAIDs: non-steroidal anti-inflammatory drugs, PPIs: proton pump inhibitors, H2RAs: histamine-2 receptor antagonists, SSRIs: selective serotonin reuptake inhibitors, ACE-Is: angiotensin converting enzyme inhibitors, ARBs: angiotensin receptor blockers

Of patients diagnosed with MC, 31.3% reported use of any dose of aspirin, 40.5% other NSAIDs, and 53.8% used some dose of aspirin or other NSAIDs. No significant differences in medication use were detected among newly diagnosed MC patients and those selected from the MC registry (Table 2). This contrasts with reported 14.4% aspirin, 20.3% other NSAIDs, and 28.8% use of any aspirin or other NSAIDs among patients not diagnosed with MC. Chi-squared analysis revealed significant differences between cohorts for all these medications at *p*** < **0.01. In addition, ACE-Is were used significantly less among MC patients (3.8%) than controls (12.7%) at *p* < 0.05. Use of PPIs, H2RAs, SSRIs, statins, and ARBs varied between the MC and no MC cohorts, however, no differences were statistically significant (Fig. 2). Among the subtypes of MC, use of aspirin (31.7% vs. 23.3%), other NSAIDs (68.4% vs. 31.7%), and any NSAIDs (78.9% vs. 46.7%) was significantly greater among patients with CC (*p* = 0.01). Compared to patients with no MC, these medications were more commonly used in patients with both CC and LC. Use of aspirin, other NSAIDs, or any NSAIDs was significantly higher in patients with CC than those with no MC (*p* < 0.01) and any NSAID use was significantly higher in LC compared to no MC (*p* = 0.02). No other significant differences were observed in medication use among MC subtypes.

**Table 2:** Medication usage

Medication	Total (%)	New MC (%)	MC Registry (%)	*p*-val^a^	MC (%)	No MC (%)	*p*-val^b^
Any Dose Aspirin	42 (21.21)	6 (28.57)	19 (32.20)	0.77	25 (31.25)	17 (14.41)	< 0.01
Other NSAIDs	56 (28.28)	9 (42.86)	23 (39.65)	0.80	32 (40.51)	24 (20.34)	< 0.01
Any Aspirin or NSAIDs	77 (38.89)	11 (52.38)	32 (54.24)	0.88	43 (53.75)	34 (28.81)	< 0.01
PPIs	48 (24.24)	4 (19.05)	14 (23.73)	0.66	18 (22.50)	30 (25.42)	0.64
H2RAs	10 (5.05)	1 (4.76)	5 (8.47)	0.59	6 (7.50)	4 (3.39)	0.20
SSRIs	57 (28.79)	6 (28.57)	21 (35.59)	0.56	27 (33.75)	30 (25.42)	0.20
Statins	54 (27.27)	5 (23.81)	17 (28.81)	0.66	22 (27.50)	32 (27.12)	0.95
ACE-Is	18 (9.09)	1 (4.76)	2 (3.38)	1.00	3 (3.75)	15 (12.71)	0.03
ARBs	16 (8.08)	2 (15.38)	3 (5.08)	0.21	5 (6.94)	11 (11.11)	0.36

Medication use was compared between sources of microscopic colitis (MC) patients (*p*-val^a^), and between MC and no MC (*p*-val^b^) utilizing univariate statistics. NSAIDs: non-steroidal anti-inflammatory drugs, PPIs: proton pump inhibitors, H2RAs: histamine-2 receptor antagonists, SSRIs: selective serotonin reuptake inhibitors, ACE-Is: angiotensin converting enzyme inhibitors, ARBs: angiotensin receptor blockers

Subgroup analysis was performed to assess consistency of these results between the individual groups of MC patients (Table 3). Both MC cohorts were independently associated with increased use of non-aspirin NSAIDs (new *p* = 0.03, registry *p* < 0.01) and any NSAIDs (new *p* = 0.03, registry *p* < 0.01). Additionally, the MC registry group was associated with significantly greater use of any dose of aspirin (*p* < 0.01).

**Table 3:** Subgroup analysis

Medication	New MC vs. No MC		Registry MC vs. No MC	
	Odds Ratio (95% CI)	*p*-val	Odds ratio (95% CI)	*p*-val
Any dose aspirin	2.36 (0.78–7.21)	0.11	2.80 (1.25–6.02)	< 0.01
Other NSAIDs	2.91 (1.04–7.87)	0.03	2.56 (1.26–5.16)	< 0.01
Any aspirin or NSAIDs	2.70 (1.04–7.36)	0.03	3.02 (1.55–6.07)	< 0.01
PPIs	0.69 (0.20–2.15)	0.53	0.91 (0.42–1.92)	0.81
H2RAs	1.42 (0.06–11.59)	0.56	2.62 (0.68–10.73)	0.16
SSRIs	1.17 (0.41–3.38)	0.76	1.62 (0.81–3.29)	0.16
Statins	0.84 (0.27–2.53)	0.75	1.09 (0.52–2.25)	0.81
ACE-Is	0.35 (0.02–2.44)	0.47	0.24 (0.04–1.08)	0.06
ARBs	1.45 (0.21–6.96)	0.64	0.43 (0.10–1.58)	0.25

*P*-values and odds ratios (OR) were calculated comparing rate of medication use among the two groups of microscopic colitis (MC) patients independently compared to the no-MC group to assess homogeneity of findings. 95% CI: 95% Confidence Interval, NSAIDs: non-steroidal anti-inflammatory drugs, PPIs: proton pump inhibitors, H2RAs: histamine-2 receptor antagonists, SSRIs: selective serotonin reuptake inhibitors, ACE-Is: angiotensin converting enzyme inhibitors, ARBs: angiotensin receptor blockers

Multivariate regression analysis was used to control for differences in age and gender between MC and no MC cohorts. Use of aspirin, other NSAIDs or both were found to be significant independent predictors of MC with odds ratios (OR) and 95% confidence intervals (CI) of 2.65 (1.28–5.64), 2.71 (1.42–5.28), and 3.04 (1.655.69) respectively. Furthermore, use of ACE-Is remained associated with a decrease in odds of MC with OR 0.17 (0.04–0.57). PPIs, H2RAs, SSRIs, statins, and ARBs were not significant multivariate parameters for MC (Table 4).

**Table 4:** Multivariate regression analysis

Medication	Odds ratio (95% confidence interval)	*p*-val
Any dose aspirin	2.65 (1.28–5.64)	0.01
Other NSAIDs	2.71 (1.42–5.28)	< 0.01
Any aspirin or NSAIDs	3.04 (1.65–5.69)	< 0.01
PPIs	0.69 (0.34–1.39)	0.31
H2RAs	1.94 (0.52–7.95)	0.33
SSRIs	1.22 (0.64–2.33)	0.54
Statins	0.83 (0.41–1.68)	0.61
ACE-Is	0.17 (0.04–0.57)	< 0.01
ARBs	0.43 (0.12–1.35)	0.16

Multivariate binomial logistic regressions were utilized to control for the effects of age and gender on the association between each medication and microscopic colitis diagnosis. NSAIDs: non-steroidal anti-inflammatory drugs, PPIs: proton pump inhibitors, H2RAs: histamine-2 receptor antagonists, SSRIs: selective serotonin reuptake inhibitors, ACE-Is: angiotensin converting enzyme inhibitors, ARBs: angiotensin receptor blockers

## Discussion

In this study involving a hybrid cohort of patients presenting for colonoscopy or enrolled in an established MC registry, current NSAID use was associated with a diagnosis of MC. The diagnostic yield of MC in our prospective cohort was 14.6% and was consistent with prior studies evaluating yield of colonoscopy in patients with chronic watery diarrhea [8, 23, 24]. To minimize recall bias within our registry cohort, only patients diagnosed within 3 months of their data collection were included. Importantly, medication usage was similar in both MC cohorts. In contrast to a number of recent studies, including one from our institution, this study did not find an association between MC and smoking [11–15]. This finding may reflect a smaller sample size for this study or may suggest a possible association between smoking and general diarrhea rather than explicit MC, and warrants further investigation.

Of the drugs implicated in medication induced MC, the strongest evidence is for aspirin and other NSAIDs. Since the 1980s, case reports began to suggest a linkage between NSAIDs, colonic inflammation, and CC in particular [25–27]. In addition, small case reports looking at clinical symptoms and/or biopsies after withdrawal and re-challenge have been reported, although not rigorously examined [27, 28]. In our study, all NSAID subjects were on non-selective Cyclooxygenase (COX) inhibitors. Thus, we were unable to comment on differences in selective COX-2 inhibition and the development of MC, but different patterns of COX inhibition may help us to understand the differences between studies looking at MC and type of NSAID usage.

Since then, a number of studies have further investigated this subject [29]. Both prospective and retrospective studies from Spain, Denmark, Netherlands, and the United Kingdom have found NSAIDs (OR 1.43–2.90), PPIs (OR 2.03–7.30) and SSRIs (OR 1.77–37.70) to be significant independent risk factors for MC [12, 16–18, 21]. However, the findings were not consistent across these larger studies and were subject to some limitations. Although utilization of national databases provides a large sample size and representation of an entire population, the measurement of medication exposure may be compromised. Utilizing prescription fill history fails to account for the prevalence of over-the-counter medication use, especially for medications as common as PPIs, H2RAs, or NSAIDs [30]. Further, many of the studies investigating medications and MC have utilized control groups consisting of individuals from the general population. The presence of chronic diarrhea between exposure and control groups serves as a source of potential recall bias, especially when investigated retrospectively. Patients that present with diarrhea are more likely to remember the specific environmental factors or medication exposures associated with its onset [31].

Recent studies have focused on control patients presenting with chronic watery diarrhea and biopsies negative for MC. In a retrospective case–control study of patients over a 10-year period, Zylberberg et al. [20] identified an association between MC and NSAIDs and an inverse association between MC and PPIs, H2RAs, and oral diabetes medications. However, in a prospective cohort of patients, Sandler et al. [19] did not identify any significant relationship between MC and medications. Our study prospectively identified control patients presenting with chronic diarrhea, and medication use was gathered from all patients with and without MC by our investigators around the time of diagnosis.

Based on our findings, we conclude that NSAIDs are an independent risk factor for the development of MC. However, our findings do not indicate that PPIs are a risk factor for MC. In this cohort, PPIs were used less frequently among patients recently diagnosed with MC and this difference was not significant (OR 0.69, 95% CI: 0.34–1.39). Compared to previous studies, PPIs were used less frequently among patients with MC and more frequently among controls [12, 16, 18, 32]. Importantly, use of PPIs is a risk-factor for diarrhea and therefore the control group used in this study may have had a greater proportion of PPI users than previously described non-diarrheal controls [33].

Our study also found an inverse association between MC and ACE-I use (OR 0.17, 95% CI: 0.04–0.57), which should be interpreted with caution given the small sample size. The findings of Masclee et al. [16] suggested a significant association between ACE-Is and MC (OR 2.5, 95% CI: 1.5–4.2), however, this may have reflected the association between ACE-Is and rate of undergoing colonoscopy. Taken together, these results suggest a need for further study of the relationship between ACE-Is and MC.

While NSAIDs are well known to cause damage throughout the gastrointestinal tract, the precise mechanism by which NSAIDs promote the development of MC is largely unclear. Prior studies have suggested that inhibition of intestinal prostaglandin synthesis due to the inactivation of cyclooxygenase may play a role, leading to increased intestinal permeability [34–37]. This may lead to further mucosal disruption and activation of pericryptal fibroblasts, leading to thickening of the collagen table as has been associated with NSAID use [38]. NSAIDs may also alter the intestinal microvasculature, with endothelial dysfunction being a contributor to MC pathogenesis. Thus, the role of NSAIDs leading to a diagnosis of MC is biologically plausible [27]. However, whether NSAIDs cause histologic changes mimicking MC, or lead to the development of true MC is unclear.

Despite this, it must be considered that many of the drugs implicated in medication induced MC have known side-effects of diarrhea [33]. Studies to date are unable to ascertain whether medications are causal in the development of MC, or trigger symptomatic flare in susceptible patients with undiagnosed MC. Although a universal approach for measurement of adverse reactions to medication has not been adopted, there is a clear need for high quality trials utilizing challenge, withdrawal, and re-challenge procedures to understand the histologic changes associated with MC and medication use [39].

The principal strengths of this study are its prospective collection of medication usage prior to colonoscopy and use of a control group that presented with diarrhea. There are several limitations to our study. All participants were recruited from a single center serving a predominantly Caucasian community which may limit generalizability. Further, hybridizing our prospective cohort with patients enrolled in an institutional MC registry may have introduced some recall bias. However, registry data was collected within three months of MC diagnosis, and it did not significantly differ from that collected from prospectively enrolled patients with MC. We feel that this data is a more accurate collection of over-the-counter medication use than a purely retrospective chart review and strengthens our results and conclusions. Lastly, dosage and duration of medications used by study subjects were variable, and did not allow for the determination of potentially critical dosages or temporal associations involved in drug induced MC.

## Conclusions

We found that chronic use of NSAIDs, but not other previously implicated medications, was independently associated with the development of MC. This data strengthens the assertion of the role of NSAIDs in most inflammatory colitides. Further studies utilizing withdrawal and re-challenge are needed to determine the effects of NSAID discontinuation on disease progression and assess true causality. Additionally, future investigation is needed to examine the effects of NSAIDs on the natural history of MC such as budesonide dependence or severity of presentation.

## Supplementary Information

**Additional file 1**. Medications and microscopic colitis questionnaire. Script used to survey patients on their medication usage prior to colonoscopy evaluation of chronic diarrhea.

## Data Availability

The datasets used and/or analyzed during the current study are available from the corresponding author on reasonable request.

## Abbreviations

MC: Microscopic colitis
LC: Lymphocytic colitis
CC: Collagenous colitis
NSAIDs: Non-steroidal anti-inflammatory drugs
PPIs: Proton-pump inhibitors
SSRIs: Selective serotonin reuptake inhibitors
ACE-Is: Angiotensin-converting enzyme inhibitors
ARBs: Angiotensin receptor blockers

## References

[1] Fumery M, Kohut M, Gower-Rousseau C, Duhamel A, Brazier F, Thelu F (2017). Incidence, clinical presentation, and associated factors of microscopic colitis in northern france: a population-based study. Dig Dis Sci.

[2] Gentile NM, Khanna S, Loftus EVJ, Smyrk TC, Tremaine WJ, Harmsen WS (2014). The epidemiology of microscopic colitis in Olmsted County from 2002 to 2010: a population-based study. Clin Gastroenterol Hepatol Off Clin Pract J Am Gastroenterol Assoc.

[3] Pardi DS, Loftus EVJ, Smyrk TC, Kammer PP, Tremaine WJ, Schleck CD (2007). The epidemiology of microscopic colitis: a population based study in Olmsted County. Minnesota Gut.

[4] Verhaegh BPM, Jonkers DMAE, Driessen A, Zeegers MP, Keszthelyi D, Masclee AAM (2015). Incidence of microscopic colitis in the Netherlands. A nationwide population-based study from 2000 to 2012. Dig Liver Dis Off J Ital Soc Gastroenterol Ital Assoc Study Liver.

[5] Olesen M, Eriksson S, Bohr J, Järnerot G, Tysk C (2004). Lymphocytic colitis: a retrospective clinical study of 199 Swedish patients. Gut.

[6] Williams JJ, Kaplan GG, Makhija S, Urbanski SJ, Dupre M, Panaccione R (2008). Microscopic colitis-defining incidence rates and risk factors: a population-based study. Clin Gastroenterol Hepatol Off Clin Pract J Am Gastroenterol Assoc.

[7] Agnarsdottir M, Gunnlaugsson O, Orvar KB, Cariglia N, Birgisson S, Bjornsson S (2002). Collagenous and lymphocytic colitis in Iceland. Dig Dis Sci.

[8] Fernández-Bañares F, Salas A, Forné M, Esteve M, Espinós J, Viver JM (1999). Incidence of collagenous and lymphocytic colitis: a 5-year population-based study. Am J Gastroenterol.

[9] Bohr J, Tysk C, Eriksson S, Järnerot G (1995). Collagenous colitis in Orebro, Sweden, an epidemiological study 1984–1993. Gut.

[10] Bohr J, Tysk C, Eriksson S, Abrahamsson H, Järnerot G (1996). Collagenous colitis: a retrospective study of clinical presentation and treatment in 163 patients. Gut.

[11] Yen EF, Pokhrel B, Du H, Nwe S, Bianchi L, Witt B (2012). Current and past cigarette smoking significantly increase risk for microscopic colitis. Inflamm Bowel Dis.

[12] Fernández-Bañares F, de Sousa MR, Salas A, Beltrán B, Piqueras M, Iglesias E (2013). Epidemiological risk factors in microscopic colitis: a prospective case-control study. Inflamm Bowel Dis.

[13] Burke KE, Ananthakrishnan AN, Lochhead P, Olen O, Ludvigsson JF, Richter JM (2018). Smoking is associated with an increased risk of microscopic colitis: results from two large prospective cohort studies of US Women. J Crohns Colitis.

[14] Vigren L, Sjöberg K, Benoni C, Tysk C, Bohr J, Kilander A (2011). Is smoking a risk factor for collagenous colitis?. Scand J Gastroenterol.

[15] Münch A, Tysk C, Bohr J, Madisch A, Bonderup OK, Mohrbacher R (2016). Smoking status influences clinical outcome in collagenous colitis. J Crohns Colitis.

[16] Masclee GMC, Coloma PM, Kuipers EJ, Sturkenboom MCJM (2015). Increased risk of microscopic colitis with use of proton pump inhibitors and non-steroidal anti-inflammatory drugs. Am J Gastroenterol.

[17] Verhaegh BPM, de Vries F, Masclee AAM, Keshavarzian A, de Boer A, Souverein PC (2016). High risk of drug-induced microscopic colitis with concomitant use of NSAIDs and proton pump inhibitors. Aliment Pharmacol Ther.

[18] Bonderup OK, Fenger-Grøn M, Wigh T, Pedersen L, Nielsen GL (2014). Drug exposure and risk of microscopic colitis: a nationwide Danish case-control study with 5751 cases. Inflamm Bowel Dis.

[19] Sandler RS, Keku TO, Woosley JT, Galanko JA, Peery AF (2021). Medication use and microscopic colitis. Aliment Pharmacol Ther.

[20] Zylberberg HM, Kamboj AK, De Cuir N, Lane CM, Khanna S, Pardi DS (2021). Medication use and microscopic colitis: a multicentre retrospective cohort study. Aliment Pharmacol Ther.

[21] Fernández-Bañares F, Esteve M, Espinós JC, Rosinach M, Forné M, Salas A (2007). Drug consumption and the risk of microscopic colitis. Am J Gastroenterol.

[22] R Core Team. R: A language and environment for statistical computing. Vienna, Austria; 2020.

[23] Ellingson D, Miick R, Chang F, Hillard R, Choudhary A, Ashraf I (2011). Diagnostic yield of microscopic colitis in open access endoscopy center. Gastroenterol Res.

[24] Kane JS, Sood R, Law GR, Gracie DJ, To N, Gold MJ (2016). Validation and modification of a diagnostic scoring system to predict microscopic colitis. Scand J Gastroenterol.

[25] Tanner AR, Raghunath AS (1988). Colonic inflammation and nonsteroidal anti-inflammatory drug administration. An assessment of the frequency of the problem. Digestion.

[26] Giardiello FM, Hansen FC, Lazenby AJ, Hellman DB, Milligan FD, Bayless TM (1990). Collagenous colitis in setting of nonsteroidal antiinflammatory drugs and antibiotics. Dig Dis Sci.

[27] Riddell RH, Tanaka M, Mazzoleni G (1992). Non-steroidal anti-inflammatory drugs as a possible cause of collagenous colitis: a case-control study. Gut.

[28] Wilcox GM, Mattia A (2002). Collagenous colitis associated with lansoprazole. J Clin Gastroenterol.

[29] Lucendo AJ (2017). Drug exposure and the risk of microscopic colitis: a critical update. Drugs RD.

[30] Ilkhanoff L, Lewis JD, Hennessy S, Berlin JA, Kimmel SE (2005). Potential limitations of electronic database studies of prescription non-aspirin non-steroidal anti-inflammatory drugs (NANSAIDs) and risk of myocardial infarction (MI). Pharmacoepidemiol Drug Saf.

[31] Grimes DA, Schulz KF (2002). Bias and causal associations in observational research. Lancet Lond Engl.

[32] Keszthelyi D, Jansen SV, Schouten GA, de Kort S, Scholtes B, Engels LGJB (2010). Proton pump inhibitor use is associated with an increased risk for microscopic colitis: a case-control study. Aliment Pharmacol Ther.

[33] Abraham B, Sellin JH (2007). Drug-induced diarrhea. Curr Gastroenterol Rep.

[34] Bjarnason I, Hayllar J, MacPherson AJ, Russell AS (1993). Side effects of nonsteroidal anti-inflammatory drugs on the small and large intestine in humans. Gastroenterology.

[35] Wallace JL (1997). Nonsteroidal anti-inflammatory drugs and gastroenteropathy: the second hundred years. Gastroenterology.

[36] Ballinger A (2008). Adverse effects of nonsteroidal anti-inflammatory drugs on the colon. Curr Gastroenterol Rep.

[37] Jenkins AP, Trew DR, Crump BJ, Nukajam WS, Foley JA, Menzies IS (1991). Do non-steroidal anti-inflammatory drugs increase colonic permeability?. Gut.

[38] Kakar S, Pardi DS, Burgart LJ (2003). Colonic ulcers accompanying collagenous colitis: implication of nonsteroidal anti-inflammatory drugs. Am J Gastroenterol.

[39] Agbabiaka TB, Savović J, Ernst E (2008). Methods for causality assessment of adverse drug reactions: a systematic review. Drug Saf.

