# Supplementary material for: Nonsteroidal anti-inflammatory drug exposure and the risk of microscopic colitis
Source: BMC Gastroenterol. 2022 Jul 30;22:367. doi: 10.1186/s12876-022-02438-z (PMC9338644; doi:10.1186/s12876-022-02438-z)
Supplement: Supplementary file 1 — Additional file 1. Medications and microscopic colitis questionnaire. Script used to survey patients on their medication usage prior to colonoscopy evaluation of chronic diarrhea. [file 12876_2022_2438_MOESM1_ESM.doc]

Medications and microscopic colitis questionnaire

| **SECTION 1:** In this section we are asking about medical conditions that you may have had that affect your colon, which is also called your large intestine. The colon is a long hollow tube of your digestive tract that starts after your small intestine and goes to your rectum. |
| --- |

1. Has a doctor ever told you that you had “microscopic colitis,” “lymphocytic colitis,” or “collagenous colitis?”

1 Yes

2 No

3 Not sure

1. For how many weeks or months have you experienced loose stool or diarrhea?

_____ (# of weeks) or _____ (# of months)

1. If diarrhea or loose stool was a problem, how many bowel movements did you have in a 24 hour period (on a typical day)? ___________(# of bowel movements in a 24 hour period)

MEDICATION USE

| **SECTION 2:** Please look at the list of medicines in each of the sections below and indicate if you are taking them REGULARY now.  By REGULARLY, we mean taking the medicine at least three times a week for at least 2 weeks.  Please consult your records if you keep a record of your medications. If you answer ‘YES,’ please circle that medication. |
| --- |

1. Baby Aspirin (81mg or less). Aspirin is usually taken for ***protection of heart problems, blood thinning, and for pain and inflammation.***

| **A.** Are you taking any of these medicines REGULARLY now? |
| --- |
| 1 Yes |
| 2 No |
| 3 Not sure |

1. Any other aspirin product (aspirin, Bayer, Excedrin) – any dose (usually 325mg)

| **A.** Are you taking any of these medicines REGULARLY now? |
| --- |
| 1 Yes |
| 2 No |
| 3 Not sure |

1. The following medicines are usually taken for ***pain and inflammation.***

| Ibuprofen (Motrin, Advil) |
| --- |
| Naproxen (Naprosyn, Alleve) |
| Diclofenac ([Voltaren](http://www.rxlist.com/script/main/art.asp?articlekey=67853)) |
| Nabumetone ([Relafen](http://www.rxlist.com/script/main/art.asp?articlekey=72069)) |
| Etodolac ([Lodine](http://www.rxlist.com/script/main/art.asp?articlekey=68791)) |
| Ketorolac ([Toradol](http://www.rxlist.com/script/main/art.asp?articlekey=70576)) |
| Celecoxib (Celebrex) |
| Indomethacin ([Indocin](http://www.rxlist.com/script/main/art.asp?articlekey=70240)) |
| Oxaprozin ([Daypro](http://www.rxlist.com/script/main/art.asp?articlekey=88743)) |

| **3A.** Are you taking any of these medicines REGULARLY now? |
| --- |
| 1 Yes |
| 2 No |

1. The next set of medicines is usually taken for ***acid reflux or stomach problems*** but might be taken for other reasons. Please indicate if you are taking any of these medicines now.

| Omeprazole (Prilosec, Zegerid) |
| --- |
| Rabeprazole Sodium (Aciphex) |
| Esomeprazole Magnesium (Nexium) |
| Pantoprazole (Protonix) |
| Lansoprazole (Prevacid) |
| Dexlansoprazole (Dexilant) |

| **4A.** Are you taking any of these medicines REGULARLY now? |
| --- |
|  |
| 1 Yes |
| 2 No |

1. The next set of medicines is also taken for ***acid reflux or stomach problems*** but might be taken for other reasons. Please indicate if you are taking any of these medicines now.

| Ranitidine (Zantac) |
| --- |
| Famotidine (Pepcid) |
| Cimetidine (Tagamet) |

| **5A.** Are you taking any of these medicines REGULARLY now? |
| --- |
|  |
| 1 Yes |
| 2 No |

1. The following medicines are often taken for ***depression,*** but might be given for other reasons. Please indicate if you are taking either of these medicines now.

| Citalopram (Celexa) |
| --- |
| Duloxetine (Cymbalta) |
| Escitalopram (Lexapro) |
| Fluoxetine (Prozac) |
| Paroxetine (Paxil) |
| Sertraline (Zoloft) |
| Venlafaxine Hydrochloride (Effexor) |

| **6A.** Are you taking any of these medicines REGULARLY now? |
| --- |
|  |
| 1 Yes |
| 2 No |

1. The medicines in this section are taken for ***lowering cholesterol***. Please indicate if you are taking any of these medicines now.

| Simvastatin (Zocor) |
| --- |
| Atorvastatin (Lipitor) |
| Lovastatin (Mevacor, Altocor) |
| Fluvastatin (Lescol) |
| Pravastatin (Pravachol) |
| Rosuvastatin (Crestor) |

| **7A.** Are you taking any of these medicines REGULARLY now? |
| --- |
|  |
| 1 Yes |
| 2 No |

1. The following medicines are usually taken for ***high blood pressure***. Please indicate if you are taking any of these medicines now.

| [Benazepril](http://www.medicinenet.com/script/main/art.asp?articlekey=782) (Lotensin) |
| --- |
| [Captopril](http://www.medicinenet.com/script/main/art.asp?articlekey=710) (Capoten) |
| [Enalapril](http://www.medicinenet.com/script/main/art.asp?articlekey=853) (Vasotec) |
| [Fosinopril](http://www.medicinenet.com/script/main/art.asp?articlekey=791) (Monopril) |
| [Lisinopril](http://www.medicinenet.com/script/main/art.asp?articlekey=862) (Prinivil, Zestril) |
| [Quinapril](http://www.medicinenet.com/script/main/art.asp?articlekey=686) (Accupril) |
| [Ramipril](http://www.medicinenet.com/script/main/art.asp?articlekey=689) (Altace) |
| [Trandolapril](http://www.medicinenet.com/script/main/art.asp?articlekey=12528) (Mavik) |

| **8A.** Are you taking any of these medicines REGULARLY now? |
| --- |
|  |
| 1 Yes |
| 2 No |

| **These are all of the questions we have. Thank you very much for your help!** |
| --- |
